# Tiny Patch, Big Value: A Small Dry Forest Patch Supports Wildlife Conservation in Guanacaste, Costa Rica

**DOI:** 10.1002/ece3.71972

**Published:** 2025-08-13

**Authors:** Trevor L. Proctor, Faridah Fatungase, Jesus A. Barquero Leiva, Francisco Javier, Chelsea E. Durr, Frank V. Paladino

**Affiliations:** ^1^ School of Life Sciences University of Hawaiʻi at Mānoa Honolulu Hawaiʻi USA; ^2^ Department of Biological Sciences Purdue University Fort Wayne Fort Wayne Indiana USA; ^3^ The Leatherback Trust Playa Grande Guanacaste Costa Rica; ^4^ Universidad Técnica Nacional Concepción Provincia de Alajuela Costa Rica

**Keywords:** camera trap, conservation, mammals, neotropics

## Abstract

Tropical dry forests are among the most threatened tropical ecosystems due to ongoing agricultural expansion and development. Despite this, small forest patches persist within fragmented landscapes, though their conservation value remains poorly understood. To evaluate the role of one such patch in supporting wildlife, we deployed camera traps across three zones (Dense Forest, Grassland, and Sparse Forest) within the Murren Reserve, a small dry forest patch on the southern coastline of Guanacaste, Costa Rica. Camera traps detected 16 vertebrate species, with opportunistic encounters adding 16 more. The most detected species included the white‐nosed coati ( *Nasua narica* ), raccoon ( *Procyon lotor* ), and white‐tailed deer ( *Odocoileus virginianus* ). Additionally, we recorded nationally endangered species, including the ocelot ( *Leopardus pardalis* ), puma ( *Puma concolor* ), and mantled howler monkey ( *Alouatta palliata* ), as well as nationally threatened species such as the spotted skunk ( *Spilogale angustifrons* ) and white‐throated magpie‐jay ( *Calocitta formosa* ). Extrapolated rarefaction curves suggest that further sampling could improve species richness estimates for the Dense Forest and Grassland. Although we did not detect a difference in species composition among zones (ANOSIM, *R* = 0.14, *p* = 0.26), a generalized linear mixed model indicated that zone explained 17% of the variation in species richness. These findings highlight the role that small dry forest patches play in conserving both threatened species and broader vertebrate communities. We also provide recommendations for future research to address current data gaps and improve long‐term monitoring in fragmented landscapes.

## Graphical abstract

This study highlights the biodiversity value of the Murren Reserve, a small tropical dry forest patch in Guanacaste, Costa Rica. Using camera traps and opportunistic observations, researchers documented 32 wildlife species, including federally endangered species like the ocelot ( *Leopardus pardalis* ) and puma ( *Puma concolor* ), as well as 73 plant species. These findings underscore the critical role of small forest patches in supporting wildlife conservation and inform management strategies for fragmented landscapes.

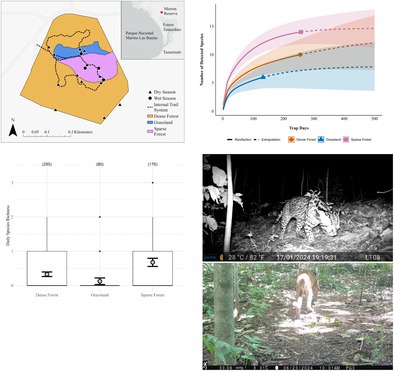

## RESUMEN

Los bosques secos tropicales se encuentran entre los ecosistemas tropicales más amenazados debido a la continua expansión agrícola y el desarrollo. A pesar de ello, pequeñas áreas de bosque persisten dentro de paisajes fragmentados, aunque su valor para la conservación sigue siendo poco comprendido. Para evaluar el papel de una de estas áreas en el soporte de la vida silvestre, colocamos cámaras trampa en tres zonas (Bosque Denso, Pastizal y Bosque Ralo) dentro de la Reserva Murren, una pequeña área de bosque seco en la costa sur de Guanacaste, Costa Rica. Las cámaras trampa detectaron 16 especies de vertebrados, y encuentros oportunistas añadieron 16 más. Las especies más detectadas incluyeron al pizote (*Nasua narica*), mapache (*Procyon lotor*) y venado cola blanca (*Odocoileus virginianus*). Además, registramos especies en peligro de extinción a nivel nacional, como el ocelote (*Leopardus pardalis*), el puma (*Puma concolor*) y el mono aullador (*Alouatta palliata*), así como especies amenazadas a nivel nacional, como la zorra manchada (*Spilogale angustifrons*) y la urraca copetona (*Calocitta formosa*). Las curvas de rarefacción extrapoladas sugieren que un muestreo adicional podría mejorar las estimaciones de riqueza de especies en el Bosque Denso y el Pastizal. Aunque no detectamos una diferencia en la composición de especies entre zonas (ANOSIM, *R* = 0.14, *p* = 0.26), un modelo lineal generalizado mixto indicó que la zona explicó el 17% de la variación en la riqueza de especies. Estos hallazgos resaltan el papel que desempeñan las pequeñas áreas de bosque seco en la conservación tanto de especies amenazadas como de comunidades vertebradas más amplias. También proporcionamos recomendaciones para futuras investigaciones que aborden vacíos de datos actuales y mejoren el monitoreo a largo plazo en paisajes fragmentados.

## 1 Introduction

Tropical dry forests, characterized by consistent warmth (> 17°C) and distinct wet and dry seasons (Dirzo et al. 2011), rank among the most endangered major tropical ecosystems (Janzen 1998). Agricultural and urban expansion continue to drive deforestation and fragmentation in these forests (Janzen 1998; Sánchez‐Azofeifa et al. 2005). Despite providing ecosystem services such as climate regulation, soil fertility, and bioregulation (Maass et al. 2005), as well as hosting high levels of endemism and carbon storage (Portillo‐Quintero et al. 2015), tropical dry forests have declined drastically in recent decades (Miles et al. 2006). In Costa Rica, nearly all primary dry forests disappeared by 1961 (Sader and Joyce 1988). Although socioeconomic shifts (Calvo‐Alvarado et al. 2009) and conservation initiatives (Quesada and Stoner 2004) have facilitated some recovery in Costa Rica, these gains are not ubiquitous. For instance, Parque Nacional Marino Las Baulas (PNMLB) protects a dry coastal zone historically bordered by tropical dry and mangrove forests (Spotila and Paladino 2004). While PNMLB safeguards the Tamarindo Estuary's mangroves, ongoing development continues to fragment the surrounding tropical dry forests beyond the park's 50 m buffer zone (Spotila and Paladino 2004).

As the region's tropical dry forests decline, fragmented landscapes persist, raising questions about their role in conserving wildlife. Small habitat patches can provide critical support for species in fragmented landscapes (Lindenmayer 2019; Wintle et al. 2019). For example, studies including critically endangered rainforest species, such as orangutans (*Pongo* sp.), Sumatran tigers ( *Panthera tigris* ), and Sunda pangolins ( *Manis javanica* ), highlight the importance of small forest fragments as habitat in Southeast Asia (Weiskopf et al. 2019; Ancrenaz et al. 2021). Similarly, Costa Rica's tropical dry forests support several faunal taxa of conservation concern, including mammals (Stoner and Timm 2004), herpetofauna (Sasa and Bolaños 2004), and avifauna (Barrantes and Sánchez 2004). Much of the Costa Rican dry forest research, however, has focused on relatively large areas of forest, like those found in Parque Nacional Santa Rosa and Parque Nacional Palo Verde (see Mata and Echeverría (2004) for maps showing Costa Rica's dry forest zones and national parks). It remains unclear if small tropical dry forest patches play a substantial role in supporting wildlife conservation.

To investigate the potential of small tropical dry forest patches to support wildlife conservation in Guanacaste, Costa Rica, we explored the species composition and daily species richness of medium‐to‐large vertebrates (i.e., those large enough to be detected by camera traps) within a small forest patch adjacent to PNMLB. Specifically, we conducted camera trap surveys, an effective passive wildlife monitoring technique (Wearn and Glover‐Kapfer 2019), to document species in zones of varying vegetative structure within the forest. This assessment, while not exhaustive, provides valuable insights into the species using the space as habitat.

## 2 Materials and Methods

### 2.1 Study Site

The Murren Reserve (~13 ha) is located 1.4 km inland from Playa Grande in Guanacaste, Costa Rica (ca. 10°20′36″ N, 85°50′28″ W). The reserve is bordered by farmland to the east, the Tamarindo Estuary to the south, and urban development to the north and west, and has an elevation ranging from 15 to 23 m above sea level. The region has a tropical dry climate, receiving approximately 1470 mm of rain between May and October and 130 mm of rain between November and April, while maintaining a median temperature of about 27°C–29°C year‐round (https://www.imn.ac.cr/en/inicio).

A simple fence surrounds the reserve to prevent disturbance by grazing cattle and other domestic animals and to discourage anthropogenic disturbance. However, unauthorized people and domesticated animals occasionally manage to make it into the reserve. Additionally, the property owner (The Leatherback Trust) maintains a circuit‐type trail used for guided educational tours and a perimeter trail for maintenance of the fence, which contributes to some disturbance.

**FIGURE 1:**
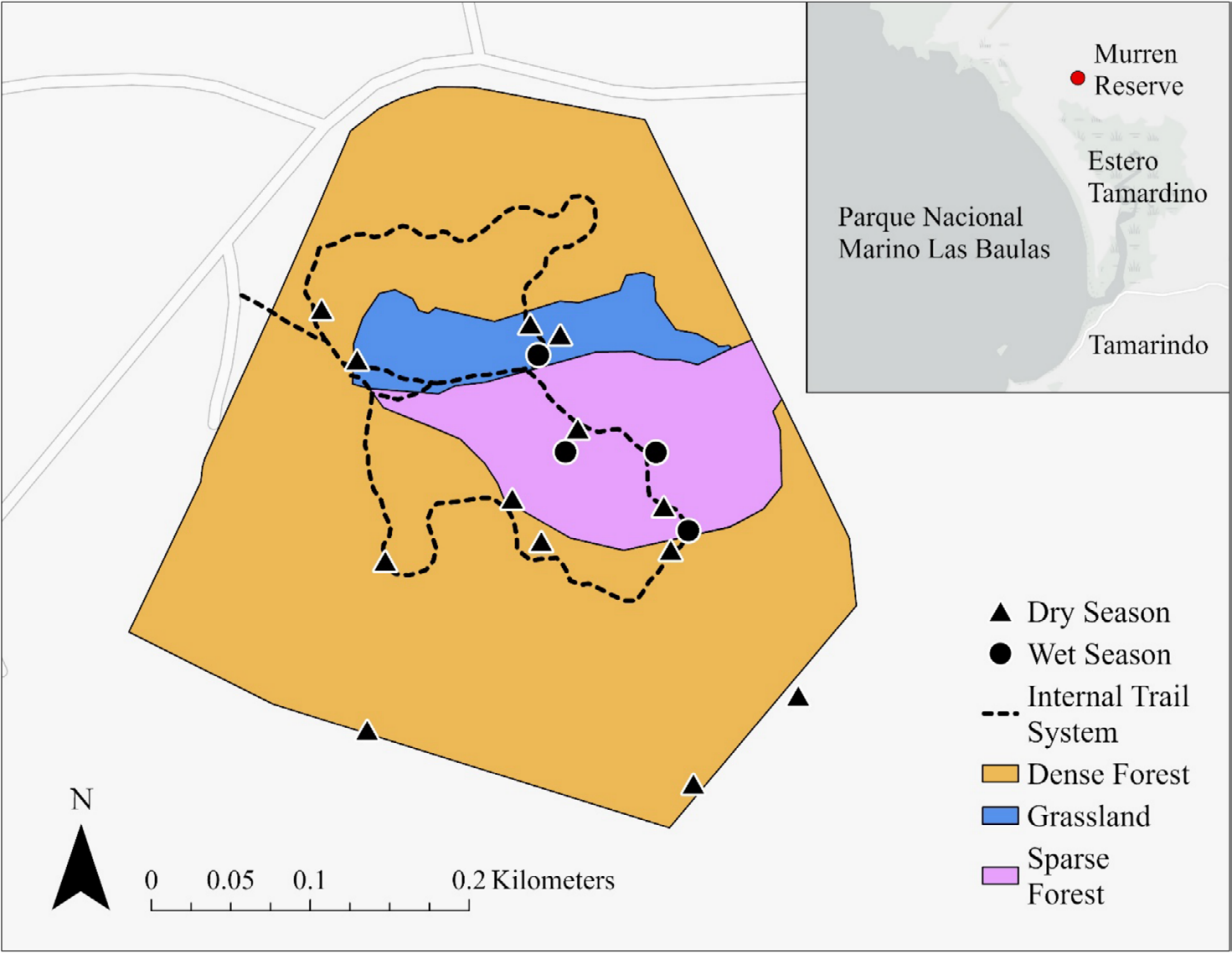
Map of the Murren Reserve, Guanacaste, Costa Rica. The inset map (top right) shows the location of the Murren Reserve (red dot) relative to Parque Nacional Marino Las Baulas's Estero Tamarindo (Tamarindo Estuary), located north of the city of Tamarindo, Costa Rica. Camera traps were placed nearby the hiking trail in the Dense Forest, Grassland, and Sparse Forest zones during the 2024 dry and wet seasons.

**FIGURE A1:**
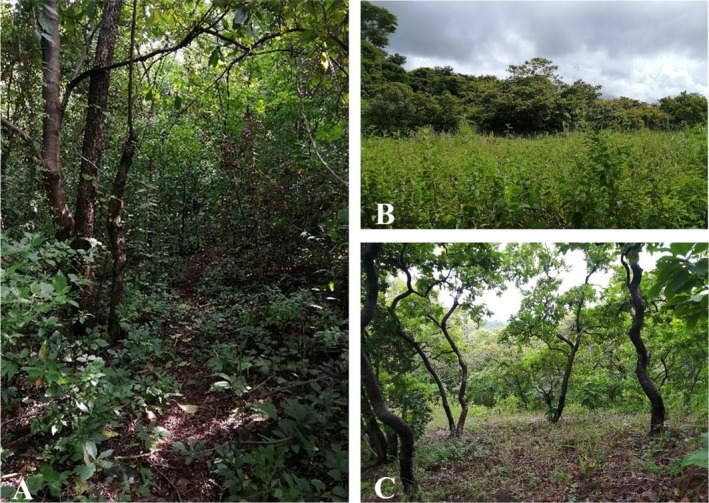
Photographic examples of the vegetative structure of the (A) Dense Forest, (B) Grassland, and (C) Sparse Forest.

To characterize the Murren Reserve, we developed a map (using ArcGIS Pro 3.4.0) based on the World Vegetation Classification System (UNESCO 1973; Müeller‐Dombois and Ellenberg 1974). Additionally, we considered the cavity fraction covered (CCF) using satellite images from previous and recent years and ground‐truthed the information in the field (Kappelle et al. 2002). In doing so, we identified three unique zones within the tropical dry forest patch: a Dense Forest (10.78 ha), a Grassland (0.94 ha), and a Sparse Forest (2.09 ha; Figure 1; see Figure A1 for photographs of vegetative structure). Additionally, we opportunistically recorded plant species in each of the three zones to compile a preliminary list of flora, including trees, shrubs, and other vascular plants (Table A1). However, we note that this was not a systematic vegetation survey, so sampling effort and identification methods varied and were not standardized. To trace the route of the trails throughout the reserve, we used a satellite locator (GNSS) to mark waypoints every 5 m.

### 2.2 Data Collection

Because the number of observations and species detected varied depending on location and season during a previous study in northern Guanacaste (Yaney‐Keller et al. 2022), we placed motion‐sensor game cameras at 13 sites throughout the three zones during the dry season (P60 Trail Camera, Meidase; January 8–March 7, 2024; mean = 38 ± 21 days [SE]) and at four sites between the Grassland and Sparse Forest during the wet season (CORE DS‐4K, Bushnell, and unknown model, Browning; June 28–August 30, 2024; mean = 29 ± 4 days) in 2024. To maximize the number of observations, we placed cameras near trails in locations thought to have moderate‐to‐high amounts of wildlife movement (based on previous sightings, naturally formed trails, and openings in the vegetation). We placed a single camera at each location, strapped to trees (about 30 cm high from the ground), facing the direction of suspected animal traffic. We programmed the cameras to capture 10–30 s videos with low or medium sensitivity (depending on the amount of vegetation in front of the camera). We checked the SD cards and batteries of each camera trap during site visits and replaced them as needed, noting that we experienced no known camera malfunctions throughout the duration of this study. Additionally, during our fieldwork, we noted opportunistic observations of vertebrates in the reserve, using direct sightings and vocalizations (Table 1).

**TABLE 1:** Species accounts of vertebrates at the Murren Reserve, Guanacaste, Costa Rica, observed by the authors during 2023 and 2024.

Scientific name	Common name	Method	Zone	Costa Rica	IUCN	CITES
Aves						
*Buteogallus anthracinus*	Common black hawk	OO	G		LC **↓**	II
*Calocitta formosa*	White‐throated magpie‐jay	OO		TH	LC	
*Campylorhynchus rufinucha*	Veracruz wren	OO			LC	
*Caracara plancus*	Crested carcara	OO			LC	II
Columbidae sp.	Dove/pigeon species	CT	DF			
*Coragyps atratus*	Black vulture	OO	G		LC **↑**	
*Crotophaga sulcirostris*	Groove‐billed ani	OO	G		LC **↑**	
*Crypturellus cinnamomeus*	Thicket tinamous	CT	SF		LC **↓**	
*Dendrocincla* sp.	Woodcreeper species	OO				
*Eumomota superciliosa*	Turquoise‐browed motmot	OO			LC **↓**	
*Hylatomus lineatus*	Lineated woodpecker	OO			LC **↑**	
*Icterus pectoralis*	Spot‐breasted oriole	OO			LC **↓**	
*Piaya cayana*	Squirrel cuckoo	OO			LC **↓**	
*Rupornis magnirostris*	Roadside hawk	OO			LC **↑**	II
*Trogon melanocephalus*	Black‐headed trogon	OO			LC **↓**	
*Turdus grayi*	Clay‐colored thrush	OO, CT	SF		LC	
*Zenaida asiatica*	White‐winged dove	OO			LC **↑**	
Mammalia						
*Alouatta palliata*	Mantled howler monkey	OO		EN	VU **↓**	I
*Canis latrans*	Coyote	CT	DF, G, SF		LC **↑**	
*Cebus imitator*	White‐faced capuchin	CT			VU **↓**	
*Dasypus novemcinctus*	Nine‐banded armadillo	CT	DF, G, SF		LC	
*Didelphis marsupialis*	Southern opossum	CT	DF, G, SF		LC	
*Herpailurus yagouaroundi*	Jaguarundi	OO			LC **↓**	I
*Leopardus pardalis*	Ocelot	CT	SF	EN	LC **↓**	I
*Nasua narica*	White‐nose coati	CT			LC **↓**	III
*Odocoileus virginianus*	White‐tailed deer	CT, OO			LC	
*Procyon lotor*	Racoon	CT	DF, G, SF		LC **↑**	
*Puma concolor*	Puma	CT	SF	EN	LC **↓**	I
*Sciurus variegatoides*	Variegated squirrel	CT, OO			LC	
*Spilogale angustifrons*	Spotted skunk	CT	DF, G, SF	TH	LC	
Reptilia						
*Ctenosaura similis*	Black Iguana	CT	SF		LC	II

*Note:* The table shows the method of observation (CT, camera trap; OO, opportunistic observation [audio or visual]), zone (DF, Dense Forest; G, Grassland; SF, Sparse Forest), status in Costa Rica (EN, endangered; TH, threatened), IUCN Red List assessment (*↓*, decreasing trend; *↑*, increasing trend; LC, least concern; VU, vulnerable), and CITES protection (I, II, or III).

We manually sorted videos, filtering out those with either no or unidentifiable animals. When there was uncertainty about the identification of an animal, two or more authors checked the video to ensure proper identification. Videos of domesticated animals (cows, horses, and dogs) or humans were removed before analysis. To maintain consistency with Yaney‐Keller et al. (2022), we used 60 min independence filters, considering an observation to be a unique capture event (UCE) if the species in the video had not been observed by the same camera within the last 60 min. In cases where multiple individuals could be identified within a single video, each individual was considered a UCE. If two videos captured multiple individuals within 60 min of one another, we selected the video with the larger number of identifiable individuals to count UCEs (noting that we did not record the identification of the individuals). We logged the date and time recorded in each video; however, the dates and times of cameras placed during the wet season were set incorrectly and could only be used for identifying UCEs. Therefore, we only considered UCEs from the dry season in most of our analyses (i.e., the generalized linear mixed model, analysis of similarity, and non‐metric multidimensional scaling plot described below). Wet season detections were only used to report the total UCE counts per species and to create the rarefaction curves, as described below.

### 2.3 Data Analysis

We used R programming software V 4.4.2 (R Core Team 2024) to conduct our analyses. To assess our sampling effort, we used the “iNEXT” (Chao et al. 2014; Hsieh et al. 2024) and “ggplot2” packages (Wickham 2016) to create rarefaction curves for each zone. Using trap days as the sampling unit (defined as a 24 h period per camera), we extrapolated the curves to 500 days, bootstrapping 1000 times with a 95% confidence interval.

To compare the daily species richness between zones during the dry season, we used the “glmmTMB” package (Brooks et al. 2017) to create a generalized linear mixed model (GLMM), using a Poisson distribution with a log link function. We used daily species richness as the response variable, zone as a fixed effect, and camera as a random effect. We calculated an overdispersion ratio of 0.73, indicating that the Poisson distribution was appropriate. Residual diagnostics and zero‐inflation tests revealed no strong violations of model assumptions. We used the “DHARMa” package (Hartig 2024) to simulate residuals and plotted an autocorrelation function (ACF) to assess temporal autocorrelation at the landscape level. Although the lag‐1 autocorrelation slightly exceeded the 95% confidence interval, the plot did not suggest sustained temporal autocorrelation. We used profile likelihood to calculate the coefficients' 95% confidence intervals (CIs). We also calculated the mean daily species richness for each zone and their corresponding 95% CIs. To compare the observed species composition, we used the package “vegan” (Oksanen et al. 2025) to calculate the Jaccard dissimilarity indices between each camera, created a dissimilarity matrix, and ran an analysis of similarity (ANOSIM) with cameras grouped by zone. We also created a non‐metric multidimensional scaling plot with convex hulls to visualize dissimilarity.

Additionally, we created a stacked bar chart showing the total number of unique capture events per species at each zone (using the “tidyverse,” “ggpattern,” “gridExtra,” and “grid” packages; Auguie 2017; Wickham et al. 2019; Fc and Davis 2024). We checked if the observed wildlife was listed by CITES (Convention on International Trade in Endangered Species of Wild Fauna and Flora), along with their regional (La Gaceta Diaro Oficial 2017) and global (IUCN 2024) status (Table 1).

## 3 Results

**FIGURE 2:**
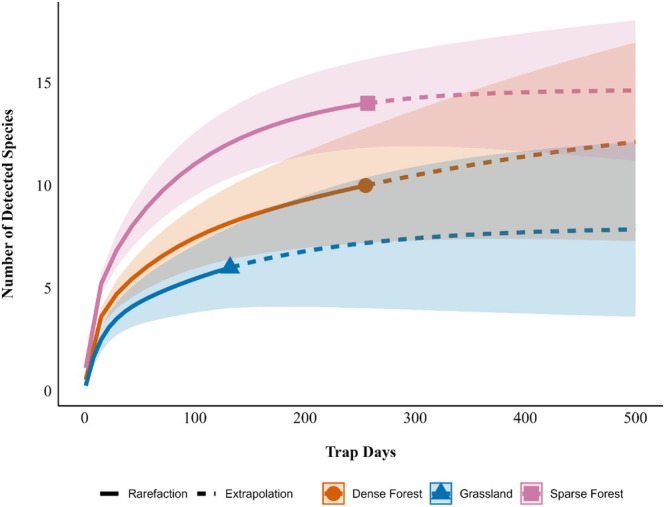
Extrapolated rarefaction curves of the number of detected species at the three described zones within the Murren Reserve in Guanacaste, Costa Rica, during 2024.

Rarefaction curves suggest that we likely observed most of the detectable vertebrates (i.e., terrestrial vertebrates large enough to be detected by a trail camera) present in the Sparse Forest, but greater trapping effort may be necessary to detect additional species in the Dense Forest and Grassland (Figure 2).

**FIGURE 3:**
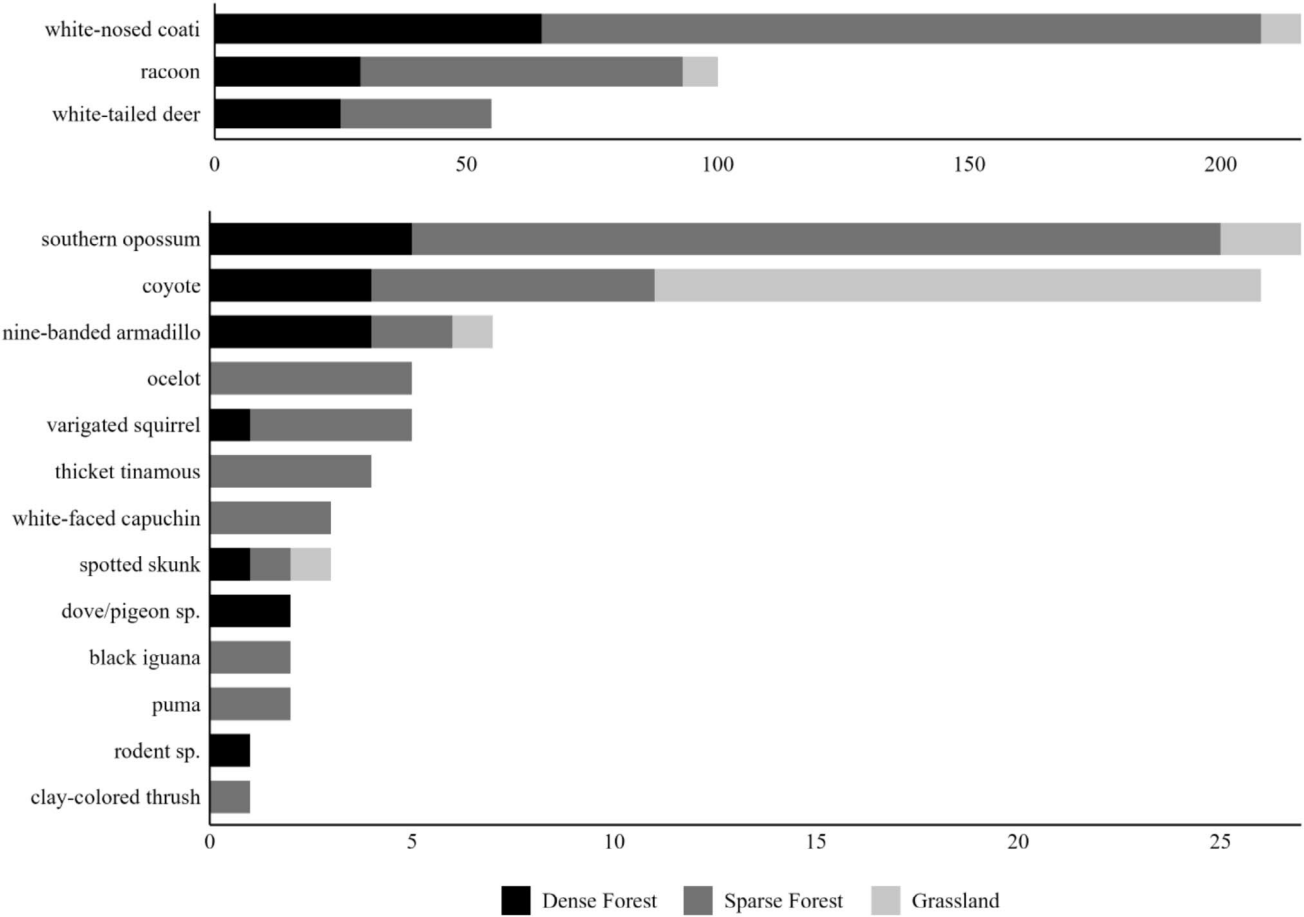
Number of unique capture events per species, captured by trail cameras, within the Murren Reserve in Guanacaste, Costa Rica. See Table 1 for corresponding scientific names.

**FIGURE 4:**
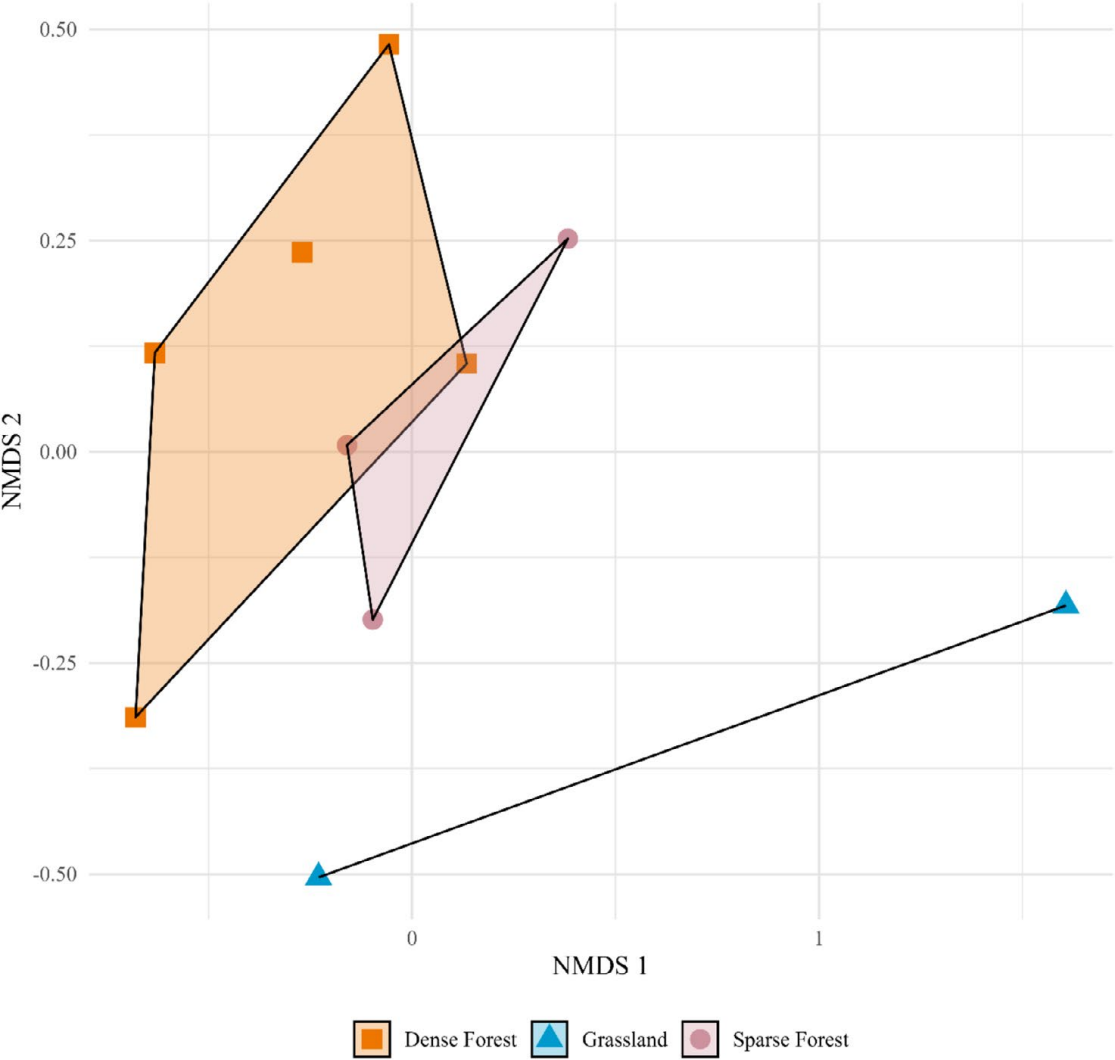
Non‐metric multidimensional scaling (NMDS) plot based on a Jaccard dissimilarity matrix illustrating differences in detected species composition among three zones (Dense Forest, Grassland, and Sparse Forest) during the 2024 dry season within the Murren Reserve in Guanacaste, Costa Rica.

Over 644 trap days (Dense Forest = 255≈24 days * ha^−1^, Grassland = 132≈140 days * ha^−1^, Sparse Forest = 257≈123 days * ha^−1^), our camera traps recorded 16 species total (Figure 3; with an additional 16 opportunistic species observations noted in Table 1). More specifically, our cameras detected 10 species in the Dense Forest, 6 species in the Grassland, and 14 species in the Sparse Forest (Figure 3). Of these, six species were only observed in the Sparse Forest, and one species was only observed in the Dense Forest. However, using data from the dry season (511 trap days; Dense Forest = 255≈24 days * ha^−1^, Grassland = 80≈85 days * ha^−1^, Sparse Forest = 176≈84 days * ha^−1^), we did not detect a substantial dissimilarity in species composition among zones (ANOSIM, *R* = 0.14, *p* = 0.26). Similarly, the NMDS ordination had a low stress level (0.05) and showed substantial overlap between the Dense and Sparse Forest zones (Figure 4).

**FIGURE 5:**
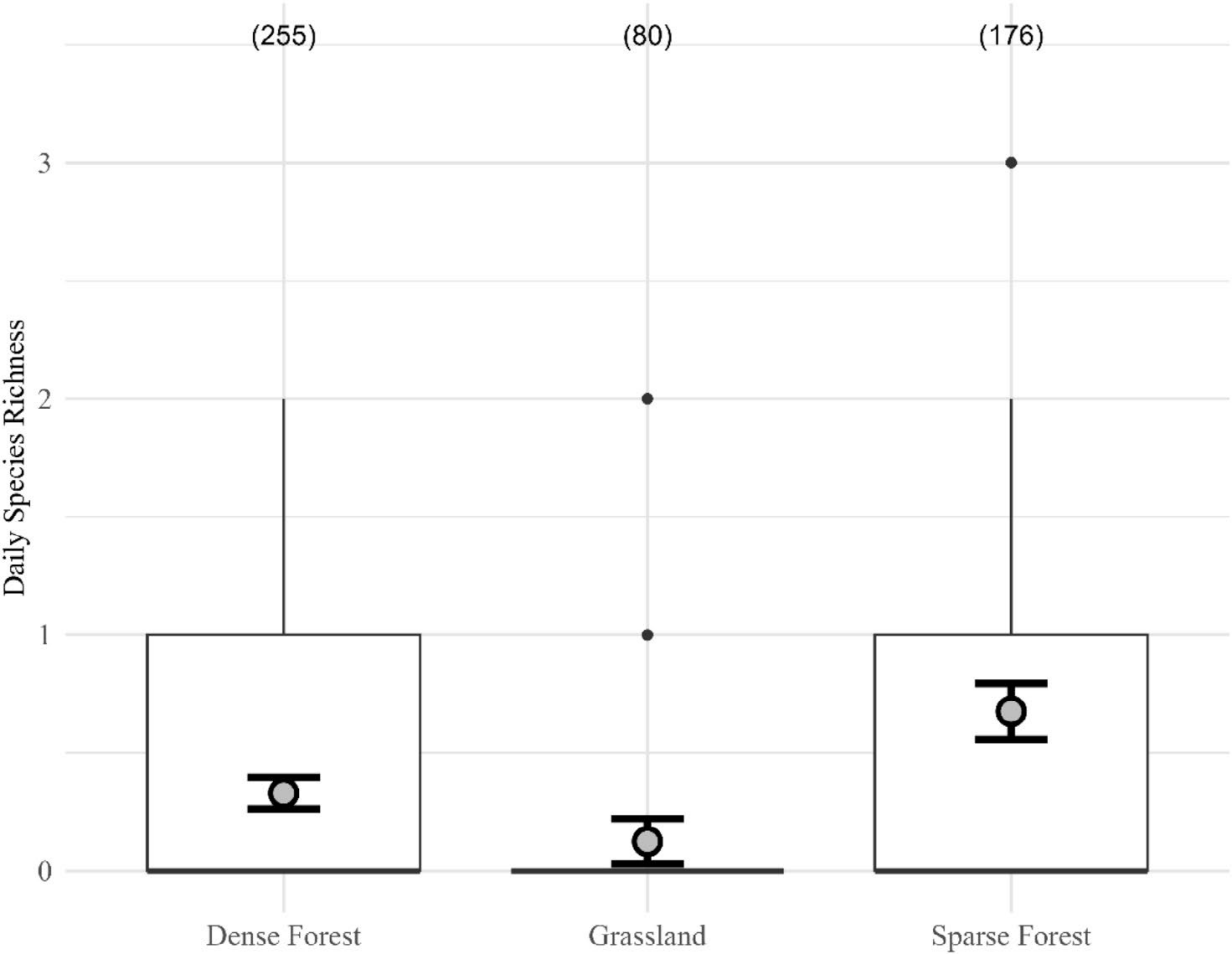
Daily species richness during the 2024 dry season at each of three zones within the Murren Reserve in Guanacaste, Costa Rica. Boxplots show the interquartile range (box), range of values (whiskers), outliers (black dots), along with the mean (gray dots) and corresponding 95% CI (bolded error bars).

Our GLMM revealed that cameras in the Grassland recorded lower daily species richness than those in the Dense Forest, while cameras in the sparse forest trended toward higher richness (although both had *p* > 0.05; Table 2). The fixed effect of zone explained 17% of the variance in species richness (marginal *R* ^2^ = 0.173), while the full model explained 53% (conditional *R* ^2^ = 0.530). Mean observed daily species richness was 0.33 (95% CI = 0.26–0.40) in the Dense Forest, 0.13 (0.03–0.22) in the Grassland, and 0.68 (0.56–0.80) in the Sparse Forest (Figure 5).

**TABLE 2:** Results of a generalized linear mixed model (GLMM) comparing daily species richness across three zones (Dense Forest, Grassland, and Sparse Forest) during the 2024 dry season in the Murren Reserve, Guanacaste, Costa Rica.

Fixed effects	Estimate (*β*)	SE	*z*	*p*	95% CI (log scale)
(Intercept)	−1.39	0.41	−3.40	< 0.001	(−2.45, −0.60)
Grassland vs. Dense Forest	−1.19	0.82	−1.45	0.147	(−3.10, 0.55)
Sparse Forest vs. Dense Forest	0.78	0.68	1.14	0.256	(−0.68, 2.49)

Random effects	Variance	SD	95% CI (variance)
Camera (Intercept)	0.87	0.93	(0.26, 3.39)

*Note:* The model used a Poisson distribution with a log link function, with zone as a fixed effect and camera as a random effect. Profile likelihood was used to calculate 95% confidence intervals.

## 4 Discussion

Our camera traps recorded 16 species of vertebrates in the Murren Reserve, with an additional 16 observed through direct sightings, for a total of 32 species. This number aligns with expectations based on similar studies. For example, Pacheco et al. (2022) documented 19 mammal species in the 70 ha Sierra Zapote Reserve, a tropical humid forest similar to dry forest ecosystems but with greater wet season rainfall. In the larger dry forests of the Guanacaste Conservation Area, Yaney‐Keller et al. (2022) detected just over 40 species, while Montalvo et al. (2023) reported 64 species, including amphibians. Although these studies detected more species, they surveyed much larger areas (300–700 ha) than the Murren Reserve (~13 ha), which likely accounts for the difference.

**FIGURE 6:**
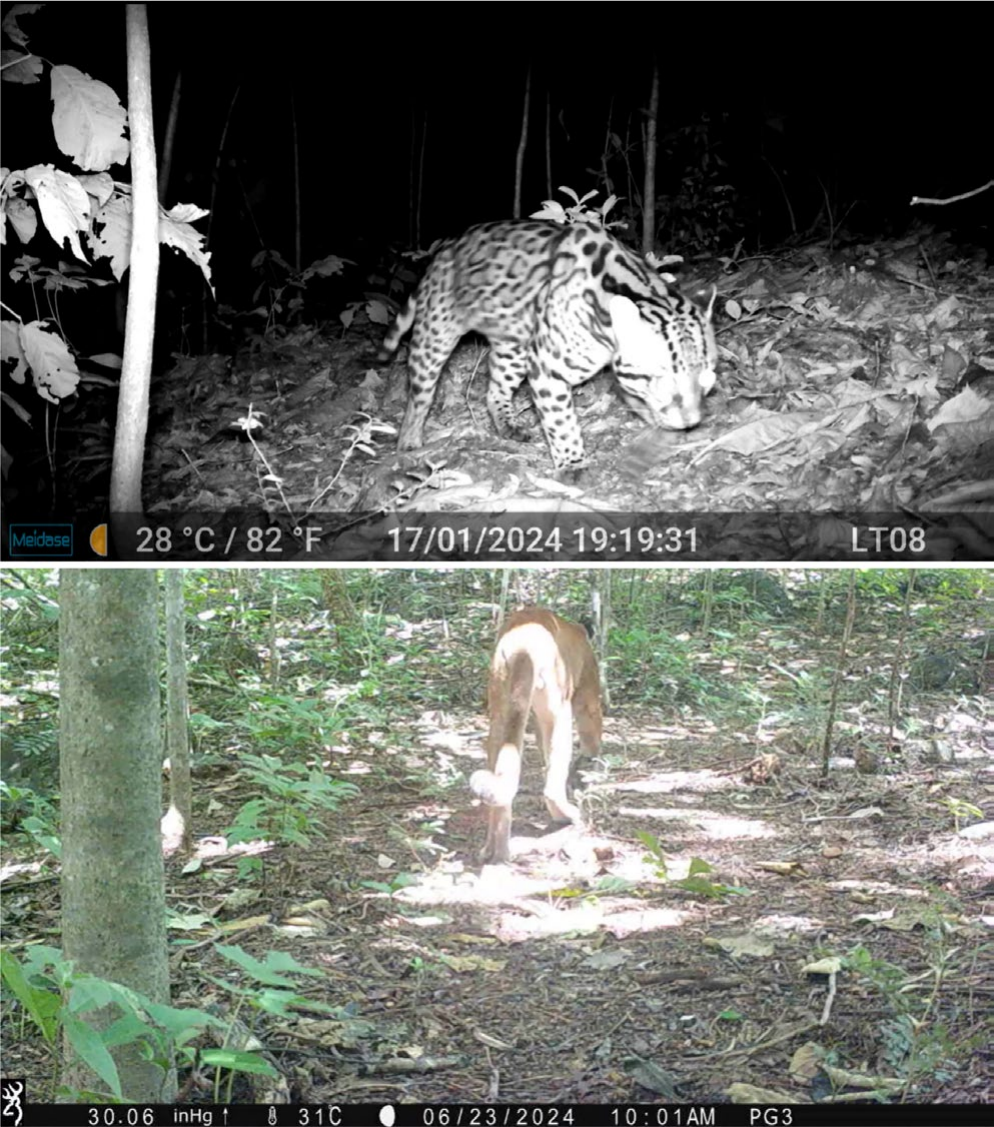
Nationally endangered ocelot (top; *Leopardus pardalis* ) and puma (bottom; *Puma concolor* ) recorded by trail cameras placed within the Murren reserve in Guanacaste, Costa Rica.

For this reason, larger tracts of land are often prioritized in biodiversity conservation (Fahrig 2003). However, small patches can also serve as critical refuges for wildlife (Lindenmayer 2019; Wintle et al. 2019). This appears true for the Murren Reserve, which supports several species of conservation concern, including the nationally endangered howler monkey ( *Alouatta palliata* ), ocelot ( *Leopardus pardalis* ), and puma ( *Puma concolor* ), as well as the nationally threatened magpie jay ( *Calocitta formosa* ) and spotted skunk ( *Spilogale angustifrons* ; Table 1). The detections of ocelots and pumas (Figure 6), species with large home ranges ( *P. concolor* : 28,180 ± 2560 ha; *L. pardalis* : 1200 ± 310 ha; Gonzalez‐Borrajo et al. 2017), could suggest that the reserve contributes to landscape connectivity. Although these species had few detections (Figure 3), both were sighted in or near the reserve during previous years (personal observation). Future research is necessary to validate the assertion that the reserve functions as a steppingstone for wide‐ranging predators. This is especially relevant to regional conservation strategies, as the Murren Reserve lies within the Costero Marino Baulas–Conchal Biological Corridor, one of 44 corridors designed to maintain connectivity across Costa Rica's protected area network. According to the National System of Conservation Areas (SINAC; *Sistema Nacional de Áreas de Conservación*), these corridors represent the country's “second most important conservation strategy” (https://www.sinac.go.cr/EN‐US/correbiolo/Pages/default.aspx). Despite this, urban and agricultural expansion continues within the corridor, likely disrupting ecosystem structure through ongoing fragmentation.

Habitat heterogeneity is often positively correlated with vertebrate diversity (e.g., species richness), particularly in areas under anthropogenic influence, due to the increased availability of niches and resources (Tews et al. 2004). During this study, we identified three distinct zones within the Murren Reserve but did not detect statistical differences in species composition among them. Although some species were observed only in the Sparse Forest (Figure 3), this could reflect limited trapping effort and lower detection probabilities in the Dense Forest and Grassland zones (Figure 4). We note, however, that no single zone included all detected species. Furthermore, the reserve's structural heterogeneity may provide diverse environmental resources that reduce competition, even among generalists, potentially increasing overall species richness. While we did not test this directly, our findings highlight the need for further research on the relationship between vegetative structure and wildlife diversity in small tropical dry forest patches.

Despite detecting a wide array of medium‐to‐large vertebrates in the Murren Reserve, we did not detect several species that are common in many Neotropical dry forests, such as the collared peccary ( *Pecari tajacu* ) and Central American agouti ( *Dasyprocta punctata* ). These species were regularly detected in similar studies (Montalvo et al. 2023; Yaney‐Keller et al. 2022), suggesting that they may have been absent or occurred at very low densities during our study. Similarly, no jaguars ( *Panthera onca* ) or tapirs ( *Tapirus bairdii* ) were detected—an expected outcome given the reserve's small size, as such wide‐ranging species typically require larger protected areas (Foerster and Vaughan 2002; Gonzalez‐Borrajo et al. 2017). These findings indicate that while the reserve serves as a refuge for a subset of the regional fauna, it does not support the full complement of species found in more extensive dry forest tracts. The absence or scarcity of habitat specialists and area‐sensitive species suggests that some ecological functions may be underrepresented. For example, collared peccaries are important seed dispersers and may contribute to forest regeneration (Beck 2005). Conversely, the presence (albeit infrequent) of apex predators like pumas and ocelots suggests the reserve may still support elements of trophic complexity, helping to maintain ecological balance (Estes et al. 2011).

Overall, this study highlights the conservation value of small tropical dry forest patches. However, we acknowledge several limitations. Trapping effort, for instance, was limited, particularly in the Dense Forest and Grassland, potentially reducing the observed species richness. Similarly, traps were placed near the trails, which may have biased detections toward species that tolerate some disturbance. The study also focused largely on the dry season and spanned less than 1 year, limiting temporal resolution. Additionally, unknown detection probabilities across species and zones prevented estimates of relative abundance, limiting insights into community structure (Sollmann et al. 2013). Moreover, camera traps primarily detect medium‐to‐large terrestrial vertebrates and select bird species but are largely ineffective for detecting other fauna (e.g., small mammals and herpetofauna).

To address these gaps, we recommend future studies:

1. Increase trapping effort and duration to assess seasonal and interannual variation (especially in relation to climate trends and anthropogenic variables) and monitor less disturbed areas of the reserve, further from the trail;
2. Estimate occurrence and detection probabilities of species in the reserve;
3. Incorporate mark‐recapture models to estimate relative abundances, using software for individual identification; and
4. Implement targeted surveys for herpetofauna (McDiarmid et al. 2012; Wilkinson 2015), small mammals (McCleery et al. 2022), birds (Ralph 1993), and arboreal species (Moore et al. 2021) to develop a more comprehensive understanding of the reserve's community structure.

Additionally, given the reserve's apparent role in supporting some wide‐ranging species, we recommend future studies include connectivity assessments to evaluate how the Murren Reserve and other small dry forest patches facilitate movement across the broader landscape.

Together, our findings suggest that, despite its small size, the Murren Reserve contributes meaningfully to regional biodiversity by providing habitat for several species of conservation concern and enhancing broader landscape connectivity. While small patches like this are not substitutes for larger contiguous dry forests, their protection is likely essential for maintaining the region's ecological integrity. Continued research is needed to further clarify these roles, but we hope our findings will inform ecologically sound decisions by government officials, land managers, stakeholders, and community members facing ongoing development pressures.

## Author Contributions

**Trevor L. Proctor:** conceptualization (equal), data curation (equal), formal analysis (lead), investigation (equal), methodology (equal), writing – original draft (lead), writing – review and editing (equal). **Faridah Fatungase:** data curation (equal), formal analysis (equal), methodology (equal), writing – original draft (supporting), writing – review and editing (equal). **Jesus A. Barquero Leiva:** data curation (equal), investigation (equal), methodology (equal), writing – review and editing (equal). **Francisco Javier:** data curation (equal), investigation (equal), methodology (equal), writing – review and editing (equal). **Chelsea E. Durr:** conceptualization (supporting), formal analysis (supporting), methodology (supporting), project administration (supporting), supervision (lead), writing – original draft (supporting), writing – review and editing (equal). **Frank V. Paladino:** conceptualization (equal), data curation (supporting), formal analysis (supporting), funding acquisition (lead), investigation (equal), methodology (equal), project administration (lead), resources (equal), supervision (lead), writing – original draft (supporting), writing – review and editing (equal).

## Conflicts of Interest

The authors declare no conflicts of interest.

## Supporting information


**Data S1:** ece371972‐sup‐0001‐supinfo.xlsx.

**Data S2:** ece371972‐sup‐0002‐supinfo.csv.

**Data S3:** ece371972‐sup‐0003‐supinfo.csv.

**Data S4:** ece371972‐sup‐0004‐supinfo.txt.

**Data S5:** ece371972‐sup‐0005‐supinfo.R.

## Data Availability

All data will be uploaded as Supporting Information S1 with the document (https://doi.org/10.5061/dryad.0000000dg).

## Appendix A

**TABLE A1:** Species accounts of flora at the Murren Reserve, Guanacaste, Costa Rica, observed by the author J.A.B.L. during 2023 and 2024.

Scientific name	IUCN status	CITES	Scientific name	IUCN status	CITES
**Liliopsida**			**Magnoliopsida (Cont.)**		
*Bactris guineensis*	LC		*Lantana camara*		
*Bothriochloa pertusa*			*Lippia alba*		
*Bromelia pinguin*			*Luehea candida*	LC	
*Cenchrus pilosus*			*Lysiloma divaricatum*	LC	
*Masdevallia nidifica*		II	*Machaerium biovulatum*	LC	
*Oryza latifolia*	LC		*Maclura tinctorea*		
*Rhynchospora contracta*			*Macroscepis pleistantha*		
*Rhynchospora nervosa*			*Malachra fasciata*		
**Magnoliopsida**			*Malvaviscus arboreus*	LC	
*Albizia niopoides*	LC		*Margaritopsis microdon*	LC	
*Alibertia edulis*	LC		*Melicoccus bijugatus*	LC	
*Anacardium excelsum*	LC ↓		*Mesosphaerum suaveolens*		
*Astronium graveolens*	LC		*Mimosa camporum*		
*Bauhinia glabra*			*Neptunia plena*	LC	
*Bauhinia ungulate*			*Persea americana*	LC	
*Bursera simaruba*	LC		*Piscidia carthagenensis*	LC	
*Byrsonima crassifolia*	LC		*Pithecellobium* sp.		
*Casearia corymbosa*	LC		*Pochota fendleri*	LC	
*Cassia grandis*	LC		*Polystemma guatemalense*		
*Ceiba pentandra*	LC		*Randia armata*	LC	
*Cenostigma eriostachys*	LC		*Russelia sarmentosa*		
*Cochlospermum vitifolium*	LC		*Samanea saman*	LC	
*Cordia alliodora*	LC		*Selenicereus costaricensis*		
*Crescentia cujete*	LC		*Sida cuspidata*		
*Curatella americana*	LC		*Trichilia martiana*	LC	
*Dalbergia retusa*	CR ↓	II	*Trichilia americana*	LC	
*Enterolobium cyclocarpum*	LC		*Trigonia rugosa*	LC	
*Genipa amaricana*			*Turnera scabra*		
*Gliricidia sepium*	LC		*Vachellia collinsii*	LC	
*Godmania aesculifolia*	LC		*Waltheria indica*	LC	
*Goeppertia macrosepala*			*Spondias mombin*	LC	
*Guaiacum sanctum*	NT ↓	II	*Spondias purpurea*	LC	
*Guazuma ulmifolia*	LC		*Sterculia apetala*	LC	
*Handroanthus ochracea*	LC ↓	II	*Swietenia macrophylla*	EN **↓**	II
*Helicteres guazumifolia*			*Tabernaemontana donnell‐smithii*	LC	
*Hyperbaena tonduzii*	LC		*Tecoma stans*	LC	
*Inga* sp.			*Tithonia* sp.		

*Note:* The table shows the IUCN Red List assessment (↓, decreasing trend; LC, least concern) and CITES protections (I, II, or III).
